# The role of genetic diversity, epigenetic regulation, and sex-based differences in HIV cure research: a comprehensive review

**DOI:** 10.1186/s13072-024-00564-4

**Published:** 2025-01-03

**Authors:** Punitha Letchumanan, Kumitaa Theva Das

**Affiliations:** 1 Department of Biomedical Sciences, Advanced Medical and Dental Institute, Universiti Sains Malaysia, Kepala Batas, Penang Malaysia; 2 Department of Biological Sciences and Biotechnology, Faculty of Science and Technology, Universiti Kebangsaan Malaysia, Bangi, Selangor Malaysia; 3 Institute for Research in Molecular Medicine, Universiti Sains Malaysia, Gelugor, Penang Malaysia

**Keywords:** HIV, Genetic, Epigenetic, Chromatin, Gender, ART, Latency

## Abstract

Despite significant advances in HIV treatment, a definitive cure remains elusive. The first-in-human clinical trial of Excision BioTherapeutics’ CRISPR-based HIV cure, EBT-101, demonstrated safety but failed to prevent viral rebound. These outcomes may result from the interplay of several factors. Growing evidence indicates that intricate epigenetic modifications play a major role in the persistence of HIV latency, presenting a significant barrier to eradication efforts and causing viral rebound after ART discontinuation. Current strategies to purge the latent reservoir involve LRAs that reactivate latent proviruses. However, their clinical success is hindered by the heterogeneity of HIV reservoirs and the virus’s diverse pathways. Additionally, RNA modifications like N6-methyladenosine (m^6 A) methylation influence HIV biology beyond transcriptional control, affect RNA stability, splicing, and translation, which could enhance therapeutic efficacy. The regulatory framework of chromatin dynamics is also key to understanding viral latency and reactivation, such as Vpr’s role in reactivating latent HIV by targeting HDACs. Sex-specific factors were also shown to play an important role with females, showing stronger early immune responses and higher representation among elite controllers. This review addresses the multifaceted challenges of HIV cure research, focusing on genetic diversity, epigenetic regulation, RNA modifications, chromatin remodeling, and sex-specific factors. By integrating insights into these aspects, this paper aims to advance our understanding of HIV cure strategies and highlight directions for future research.

## Background

Excision BioTherapeutics, a California-based biotech company, recently unveiled data from the pioneering first-in-human (FIH) clinical trial of a CRISPR-based HIV cure, EBT-101 (NCT05144386) [1]. This trial, which represents a significant advancement, highlighted the complexity of translating preclinical success to clinical efficacy. EBT-101 was administered intravenously using an adenovirus-associated virus vector serotype 9 (AAV9) to aviremic HIV infected adults on stable antiretroviral therapy (ART), followed by treatment interruption at Week 12 [2, 3]. Two guide RNAs were employed to excise three specific sites targeting the viral long terminal repeats (LTRs) and Gag in the latently integrated HIV genome, generating three possible deletions: 5′LTR to Gag, Gag to 3′LTR, and 5′LTR to 3′LTR. While pre-clinical studies in mice and macaques demonstrated promising curative potential, recent data presented at the 27th ASGCT meeting in Baltimore indicated that EBT-101 did not prevent viral rebound in three participants who paused ART during the Phase 1/2 trial, necessitating the resumption of ART [4].

One plausible reason as to why EBT-101 was not able to sufficiently reach its expected potential could be due to insufficient delivery of AAV9. However, the findings of the FIH prompt a thorough discussion of the complexities of HIV. Learning from previous efforts, various research underlines the relevance of epigenetics in HIV cure research. One key challenge is the establishment of HIV latency, which is significantly controlled by epigenetic modifications such as DNA methylation and histone alterations. Ye et al. (2022) demonstrated that the recruitment of transcriptional repressor complexes can promote HIV silencing in specific cell types, such as astrocytes and microglial cells [5]. These complexes create a tightly packed, inactive chromatin structure at the HIV promoter, making the viral DNA inaccessible to gene-editing tools, unless delivered through suitable vectors. Another study showed that CRISPR was effective against transcriptionally active viral particles in latently infected J-Lat 10.6 cells, but the transcriptionally inert HIV-1 DNA remained inaccessible, likely due to modified chromatin structures [6]. This presents a significant challenge to efforts aimed at reactivating and eradicating HIV from latent reservoirs. Other than epigenetics, this paper also dives into additional factors that may be crucial to future human clinical trials by examining the impact of genetic diversity on CRISPR therapies, how post-transcriptional modifications affect viral persistence, and how hormonal and immune responses across genders influence treatment outcomes.

## Evolution of antiretroviral therapy (ART) in HIV therapy

The advent of ART in late 1980s marked a significant leap in managing HIV/AIDS, which continues to pose a significant global health challenge, with ~ 39 million people living with HIV (PLHIV) and 40.4 million deaths to date [7]. In 1987, the United States Food and Drug Administration (FDA) sanctioned the usage of Azidothymidine (AZT) as an antiretroviral medication for HIV/AIDS. AZT was effective in inhibiting viral replication and disease advancement but caused significant adverse effects, including the development of resistance to this single medication [8]. In 1996, drug combinations known as highly active antiretroviral therapy (HAART) were first outlined.

Although these combinations significantly decreased mortality and transmission risk, high adherence to daily ART remained essential [9]. Long-acting formulations of cabotegravir (CAB) and rilpivirine (RPV), administered through monthly intramuscular injections, have demonstrated HIV suppression efficacy comparable to standard once-daily oral therapy [10, 11]. Additionally, Gilead’s twice-yearly lenacapavir (LEN), a promising long-acting injectable HIV medication is currently being evaluated for its efficacy in treating HIV-infected individuals and its potential as an injectable pre-exposure prophylaxis (PrEP) for HIV-negative individuals [12].

While ART reduces the viral load to undetectable levels, it does not eradicate HIV, merely forces the virus into a dormant state. Interruption of ART can result in HIV rebound in as short as a few weeks in some patients due to the reactivation of replication competent virus [1]. While the sharp increase in HIV viral load is predominantly driven by clonal expansions from the latent reservoirs, the different rates of rebound events across different subtype infections also demonstrate the importance of having a thorough understanding of HIV genetic diversity [13, 14].

## HIV genetic diversity

HIV genetic diversity can be traced to multiple cross-species transmissions of simian immunodeficiency viruses (SIVs) from nonhuman primates to humans. This zoonotic transmission led to the emergence of distinct viral groups: M (Major), O (Outlier), N (non-M, non-O), and the recent group P. The propagation of this virus contributed to the geographical distribution of genetically distinct HIV, with multiple subtypes and recombinant forms circulating globally. As of now, there are 157 HIV circulating recombinant form (CRFs) documented in the Los Alamos National Laboratory HIV sequence database with the three most prevalent HIV subtypes in PLHIV worldwide being B (54.7%), subtype C (14.7%), and CRF01_AE (7.5%) [15].

Although ART appears effective in controlling HIV infections, increasing evidence indicates that subtype-specific drug resistance mutations (DRMs) can influence treatment efficacy [16, 17]. During HIV replication, genetic mutations vary in pattern and frequency among subtypes due to genetic differences, which in turn affect the distribution of DRMs, ART effectiveness and consequently, treatment and vaccine development [18]. There is also disparity in developing effective treatments as most research focuses on Subtype B, which may not apply to regions with other prevalent subtypes. Molecular studies have shown that proteins from different HIV subtypes, notably with glycoprotein gp120 from Subtypes A and B, affect human cells differently, emphasizing the importance of investigating molecular variations between HIV subtypes for treatment development [19].

A crucial example of this was in a study with Malawian HIV subtype C-positive virologic failure patients who were either initiating or already on first-line ART. Viruses with mutations such as K101E, Y181C, and G190A displayed significantly increased resistance to nevirapine (NVP) by up to 893-fold. H221Y mutation, in combination with Y181C/H221Y and other mutations like K103N or K101Q, further increased resistance levels to NVP, up to 100-fold (K103N) or 3000-fold (K101Q). These DRMs not only heightened resistance to the prescribed antiretroviral drugs but also expanded resistance to first-line drugs such as tenofovir [20], once again stressing the importance of genetic diversity of HIV subtypes when optimizing HIV treatment.

## Epigenetics and HIV latency

HIV reverse transcribes its RNA and integrates viral DNA into the host genome. The integrated HIV-derived double-stranded DNA, known as provirus, serves as a template for all viral-derived replication components [21, 22]. In most cases, acute infection causes replication-associated cytopathic consequences that result in cell death. However, a small subset of infected long-lived resting memory T cells harbor integrated HIV DNA which persists indefinitely [23, 24]. Since latency is transcriptionally silent, but replication-competent, ART is incapable of targeting latently infected cells.

HIV latency is sustained by epigenetic changes in its promoter region, such as histone deacetylation in proximity to the promoter and DNA methylation in CpG islands, which suppresses viral transcription. HIV integration, similar to DNA transposition, can cause genetic and epigenetic alterations, notably genomic integration-induced host genomic instability, host epigenetic status changes, and altered gene transcription and splicing regulation [25]. Studies have shown that HIV integration sites can lead to aberrant host gene transcription downstream of the integration site. HIV frequently integrates into certain genes, like the BTB domain and CNC homology 2, BACH2 gene, crucial genomic loci that have been identified as key sites of clonal expansion that played a huge role in the silencing of HIV promoters [26]. As HIV integrates into the host cell genome, it relies on the cell’s own epigenetic and transcriptional machinery, including RNAP-II and p-TEFb/CDK9, to transcribe the integrated provirus [27]. In addition to contributing to the development and maintenance of latency, these modifications have the potential to impair regular cellular processes, including prevention of transcription of important immune markers and co-factors that could inhibit viral infection and Tat-dependent transcription [28, 29].

A promising approach to eliminate HIV reservoirs is the “shock and kill” strategy, which utilizes latency-reversing agents (LRAs) to reactivate latent proviruses in the presence of HAART, making them susceptible to immune responses and viral cytopathic effects. Various classes of LRAs, including histone deacetylase inhibitors (HDACis), histone methyltransferase inhibitors (HMTis), DNA methylation inhibitors (DNMTis), and protein kinase C (PKC) agonists, have been extensively studied for their potential to disrupt latency [30]. Among these, HDACis have garnered significant attention by specifically targeting cellular histone deacetylases (HDACs), which maintain HIV latency within CD4 + T-cells. By inhibiting HDACs, HDACis can potentially reverse latency by activating HIV transcription and increasing the expression of viral proteins and virions, enabling the immune system to clear infected cells or cytolysis [31].

Several examples of HDACis include panobinostat, and romidepsin. When administered, these LRAs significantly increase cell-associated unspliced HIV RNA from CD4 + T cells. While panobinostat and romidepsin enhance total and elongated HIV transcripts, they have minimal impact on polyadenylated or multiply spliced transcripts [32, 33]. In clinical trials, these agents stimulate the production of unspliced HIV transcripts, thereby increasing HIV RNA levels in infected cells. However, integrated HIV DNA, including the size of the latent reservoir, remained unchanged, making it essential to generate peptide antigens crucial for T-cell recognition and the elimination of infected cells [26, 27, 34].

PKC agonists have also shown great promise in triggering latent viral expression via NF-κB signalling [35]. PKC agonists, such as prostratin, and bryostatin stimulate transcription factors like NF-κB to bind to the HIV long terminal repeat (LTR) region and initiate transcription [36]. However, Bryostatin-1’s ability to completely eradicate latent reservoir is limited and does not affect latent HIV transcription in vivo [37]. Another challenge is the heterogeneity of latent reservoirs as evident in the range of infected cell types, along with the varying molecular mechanisms controlling latency, which likely differ between cells [38]. Despite several LRAs being developed, their development has been hampered due to the inability to reduce latent reservoirs and significant side effects [39, 40]. Valuable lessons were gained from LRAs which were effective at impacting the epigenetic pathways. However, novel strategies are still needed to induce apoptosis or manipulate cell check points to successfully eradicate cells harboring latent reservoirs [41, 42].

## RNA modifications and their impact on HIV replication

The role of epigenetic mechanisms in gene expression and cellular identity is fundamental. While these processes often lead to long-lasting changes, gene regulation extends beyond transcription [43]. Parallel to epigenetic mechanisms, RNA-level regulation, epitranscriptomics, involves over 170 chemical modifications in almost all RNA [44, 45]. The translation of RNA into proteins is further diversified through post-transcriptional modifications (PTMs) including methylation and acetylation, which are crucial for regulating gene expression and affecting protein stability. PTM-induced conformational changes influence proteins’ interacting partners and subsequent signaling cascades [46]. In the context of HIV, these alterations are critical to the viral life cycle and have a substantial impact on the treatment efficacy.

HIV infection has been shown to modify RNA methylation patterns, impacting the efficacy of treatment. One prevalent modification is N6-methyladenosine ($\:{m}^{6}A$), found abundantly on eukaryotic mRNA [47]. The addition of a methyl group to the N6 position of adenosine is facilitated by a multi-subunit methyltransferase complex known as the $\:{m}^{6}A$ writer complex [48]. In brief, $\:{m}^{6}A$ writer complexes catalyse methyl group addition to adenosine residues within a specific motif on mRNA molecules known as the DRACH motifs. Thereafter, RNA binding proteins, acting as $\:{m}^{6}A$ readers, recognize methylated residues and influence mRNA metabolism, including secondary structure, nuclear export, stability, splicing, and degradation [49].

From previous studies, 14 $\:{m}^{6}A$ modification locations were identified in HIV RNA, including coding and noncoding regions, splicing junctions, and splicing regulatory sequences. Notably, methylation of the Rev response-element (RRE) region enhanced Rev protein binding, facilitating viral RNA export from the nucleus, and boosting HIV replication. This shows that viral infection triggers a significant increase in $\:{m}^{6}A$ in both host and viral mRNAs [47].

Besides that, the presence of $\:{m}^{6}A$ within the 5’UTR of HIV RNA was discovered in multiple studies [49–51]. This 5’UTR region contains essential regulatory components required for effective HIV replication as it enables complete RNA genomes to be incorporated into progeny virions. Two DRACH motifs in the 5’UTR are of particular interest. One is located near the primer-binding site (PBS), which is necessary for initiating reverse transcription (RT). The other is located near the dimer initiation sequence (DIS), which is involved in a monomer-dimer switch that determines whether a given full-length HIV RNA is translated (monomer) or selected for packaging (dimer). When one of these two adenosine residues was mutated, p24 levels in HEK293T producer cells increased, but the virions’ ability to infect target cells decreased [52]. Furthermore, $\:{m}^{6}A$ modifications exhibit notable conservation within the HIV genome, despite its susceptibility to mutation. Only a small fraction of the nearly 250 DRACH motifs in full-length HIV RNA seem to harbor $\:{m}^{6}A$, indicating an exceptionally high specificity in these sites [53, 54]. These findings imply the critical role of $\:{m}^{6}A$ modifications in enhancing efficient virus replication.

Given this context, the presence of HIV-induced $\:{m}^{6}A$ modifications could significantly decrease the efficacy of therapies targeting HIV replication. Specifically, modifications within RRE enhance the binding of Rev protein, which is crucial for forming nuclear export complexes. Without these modifications, the efficiency of viral RNA export and subsequent replication could be reduced, potentially leading to a decrease in viral load. This could affect the effectiveness of therapies that rely on viral replication as a target. For instance, METTL3/14 knockdown, decreased $\:{m}^{6}A$ methylation at conserved adenosines A7877 and A7883 within the RRE hairpin structure. Consequently, impaired Rev protein binding to the RRE reduces the export of viral RNA from the nucleus and inhibits viral replication [47]. Thus, the absence of $\:{m}^{6}A$ modifications demonstrate the effectiveness of therapies that target viral replication, which can have implications for the pharmacodynamics and pharmacokinetics of ART drugs. Although direct evidence linking $\:{m}^{6}A$ modifications to HIV therapy are limited, studies on other viruses suggest that targeting $\:{m}^{6}A$ modifications can make drug-resistant cells more responsive to therapy and boost immunotherapeutic outcomes [55]. CRISPR technology, with its ability to precisely target specific sequences, can be utilized to manipulate $\:{m}^{6}A$ levels, thereby offering novel therapeutic interventions against HIV.

## Histone modification and its role on HIV transcription

RNA modifications are crucial for regulating HIV replication and the effectiveness of antiviral therapies. However, chromatin dynamics, which control how DNA is accessed for transcription, also play a significant role. Failure to adapt to this interplay can result in cell death, developmental defects, and disease. Adaptive cellular responses are frequently achieved through inducible changes in gene expression [56, 57]. Chromatin modification is an ideal mechanism for dynamic gene expression, allowing rapid, adaptable, and reversible changes that facilitate DNA-templated processes such as transcription, chromosome segregation, DNA replication, and repair. Examples include histone-modifying enzymes and ATP-dependent chromatin remodelers, which adjust nucleosome position and content. These chromatin modifiers are preserved throughout evolution and control a variety of processes necessary for healthy cell division and organism growth [57, 58].

In the case of HIV, chromatin remodeling is closely linked to viral latency, replication, and the effectiveness of therapy. In the latent reservoir, the provirus remains silent, making it difficult for the immune system and therapeutic interventions to detect these cells. Many studies have shown that chromatin structure and composition are key to establishing and maintaining proviral latency, as well as the reactivation potential of latently infected cells [59–61]. The status of chromatin condensation can be influenced by a variety of mechanisms, including post-translational covalent changes of histone tails and the recruitment of repressive proteins on methylated DNA [62]. Through interactions with chromatin-associated proteins and changes in DNA accessibility, these alterations affect patterns of gene expression. The latent state of proviral DNA in infected cells is maintained by a range of enzymes that change histones [63].

One such histone modification that is crucial in regulating gene expression is histone H3 lysine 9 trimethylation (H3K9me3). H3K9me3 is associated with gene repression and is involved in establishing and maintaining heterochromatin [64, 65]. H3K9me3 has been linked to the establishment of viral latency and HIV transcription suppression [59, 64]. A recent study that investigated the early events following the entry of HIV found that core histones, including the variant H3.3, as well as linker histone H1 and the post-translational H3K9me3 modification, are rapidly loaded onto unintegrated HIV DNA. In recent years, the role of unintegrated HIV DNA has become increasingly apparent. Despite no integration, Vpr is able to promote viral transcription from unintegrated DNA, resulting in a high abundance of multiply spliced transcript, an indicator of the frequency of intact virus [66–68].

This loading of core histones occurs shortly after the virus engages with nuclear pore proteins upon nuclear entry. The significance of H3.3 loading on viral DNA processing or expression remains unclear, but it is speculated that it may contribute to the low expression of these DNAs. Furthermore, the study detected high levels of the silencing mark H3K9me3 and low levels of the activation mark H3K9Ac on unintegrated HIV DNA. These modifications correlate with the observed silencing phenotype of unintegrated HIV DNAs. Therefore, the potent silencing of unintegrated HIV DNAs by chromatinization presents a major challenge for the use of non-integrating vectors in gene therapy applications [69].

Along with histone methylation, histone acetylation is also a key factor in the dynamic mechanism of chromatin remodeling. This major post-translational modification (PTM) involves the covalent addition of an acetyl moiety to the lysine ε-amino group on histone protein tails. This process is mediated by the counteracting actions of two enzymes: histone acetyltransferases (HATs) and histone deacetylases (HDACs) [33, 70]. By removing acetyl groups from histone tails, HDACs render the chromatin structure into a more compact form of heterochromatin, making the genes inaccessible for transcription and causing transcriptional repression [71]. This mechanism is especially significant because LRAs that include HDAC inhibitors (HDACis) are meant to stimulate the transcriptional activation of latent HIV [72]. Different in vitro models of HIV latency have been used to identify LRAs, yet, no single or combination of LRAs has been shown to significantly reduce the reservoir size in clinical settings [73].

This lack of efficacy may stem from the complexity of the multiple signaling pathways involved in establishing the latent reservoir including the NF-κB, MAPK/ERK, PI3K/AKT/mTOR, Wnt/β-Catenin, TGF-β, JAK/STAT, and Notch pathways, in addition to mechanisms of chromatin modification and remodeling [74–76]. Moreover, the reactivation of the entire HIV reservoir by LRAs is often incomplete due to the heterogeneous nature of the reservoirs [77, 78], which result in the limited and inconsistent reactivation spectrum by LRAs. Therefore, the heterogeneity of cells and tissues constituting HIV reservoirs, coupled with the complex molecular pathways, creates substantial challenges in the development of a single LRA capable of fully reactivating latent virus [79]. To effectively overcome the many blockages to HIV gene expression in resting CD4 + T cells, it is commonly anticipated that combinations of LRAs will be required.

Several studies have shown that hyperacetylation of core histones on the HIV long terminal repeat (LTR) is associated with active HIV genome transcription while hypoacetylation is associated with HIV latency. An interesting observation was that the reactivation of latent HIV provirus occurred with Vpr-induced degradation of HDAC1 and HDAC3 in a VprBP-dependent manner [80], implying that the virus has developed a way to combat class I HDACs to reactivate latently infected cells. Additionally, Vpr facilitates the depletion of the cellular repressor CTIP2, which supports the establishment and persistence of HIV latency post-integration in microglial cells. By counteracting this viral gene silencing, Vpr ultimately facilitates the reactivation of HIV expression in a microglia latent model [81]. Additionally, a novel recombinant zinc finger protein transactivator, ZFPb-362-VPR, significantly enhances proviral HIV transcription in latency models, indicating the potential of Vpr-guided transcriptional activation for reactivation [82]. It is worth noting that translating the enhancement of Vpr expression into a therapeutic strategy utilizing CRISPR technology could lead to more effective reactivation of latent reservoirs, potentially resulting in improved eradication strategies. Chromatin remodeling is an important element in the regulation and reactivation of HIV latency; hence a complete understanding of these events is necessary to achieve a viable cure for HIV.

## Sex-based disparities and hormonal influence in HIV research and treatment

While understanding chromatin remodeling and epigenetic mechanisms is crucial for developing effective HIV treatments, it is equally important to consider sex-based disparities and hormonal differences in HIV research and treatment outcomes. Sensitivity to hormones can be modified due to epigenetic factors modifying hormone receptor expression. Concurrently, by recruiting epigenetic regulators, hormones can influence epigenetic factors. Taken together, the interplay between epigenetics and hormonal factors can play a key role in regulating viral transcription, and understanding the differences between both sexes would shed light on HIV treatment [83].

Despite accounting for more than half (53%) of the global HIV population, women continue to be substantially underrepresented in HIV clinical trials [84]. This generates gaps in scientific understanding regarding how HIV affects the safety and efficacy of treatment in women and men. Male and female patients may have distinct risks of acquisition, rate of illness progressions and treatment responses due to physiological and hormonal variations [85]. In the past, concerns about possible fetal injury and the need to reduce variability in study results imposed by hormonal cycles led to the exclusion of women of reproductive age from clinical research studies [86]. This exclusion resulted in a huge data gap for analyzing sex-based differences in HIV transmission and progression.

Female HIV infection is distinguished by a more robust early immune response, as demonstrated by a high CD4 + T cell count, low viral load, and high CD8 + T cell activity. On the contrary, a high viral load, a decreased CD4 + T cell count, and a decreased CD8 + T cell cytotoxic function are indicative of HIV infection in males [87, 88]. Aside from that, biological differences between males and females influence HIV susceptibility. Males’ foreskin and females’ vaginal mucosal microbiomes both contain CD4 + T cells that are susceptible to HIV infection following sexual intercourse with an infected partner. However, males are more likely than females to contract HIV during a single sexual encounter with high viral particles because male CD4 + T cells express more CCR5 receptors in their foreskin. Circumcised males have a 60% lower chance of being infected due to the loss of the foreskin. This partially explains the higher viral loads seen in males during primary HIV infection [89, 90].

In addition to that, hormones that control the menstrual cycle have an impact on the cells, bacteria, and mucosal membrane structure of the female genital tract [91]. One such hormone that thickens mucosal membranes and strengthens immune responses to infections is estrogen. As a result, during estrogen-influenced menstrual phases, women are less likely to contract HIV than during progesterone-dominated periods [92]. Estrogen receptors have also been shown to inhibit HIV DNA reactivation in T cells, while receptor antagonists are capable of activating latent HIV proviruses [93]. The differences in hormone and its effects on latency is also extremely important for key populations such as transgender people on gender-affirming hormone therapy (GAHT). Interestingly, GAHT also induces a unique methylation profile, raising an additional epigenetic perspective to be taken into consideration [94]. Learning from the hormonal differences between sexes could be a powerful tool in strategies to tackle the viral reservoir.

Another contributing factor is the presence of sex chromosomes. Females have two X chromosomes, with one of them inactivated at random in each cell during the embryonic stage. However, this inactivation is not complete and errors in this process may occur; hence certain genes on the second X chromosome remain active, potentially offering an immunological benefit. It has been reported that 15–23% of X-linked genes escape inactivation [95]. Several immune-related genes, such as some toll-like receptors (TLRs), are found on the X chromosome [96]. TLRs are important pathogen-sensing receptors that respond to a variety of microbial ligands and initiate both innate and adaptive immune responses to infection. For example, TLR-7 and TLR-8, located on the X chromosome, may recognize single-stranded viral RNA (ssRNA), including HIV genomic RNA. Genes such as TLR-7 that express themselves biallelically produce larger quantities of protein, which may contribute to a stronger innate immune response [96–98]. Studies have shown that female plasmacytoid dendritic cells (pDCs) exhibit an enhanced TLR7-mediated interferon-alpha (IFN-α) response compared to male pDCs, commonly observed in the first week of HIV infection [99]. This increased immune response could contribute to the observed differences in disease progression and treatment outcomes between sexes.

## Elite controllers and the key for a functional cure

On top of everything, when exploring HIV curative avenues, it is essential to consider the unique characteristics of elite controllers. Elite controllers comprise of less than 1% of PLHIV across several multicenter cohorts, who maintain undetectable viral loads and stable CD4 + T cell counts without treatment [21, 100]. Holding the key to an HIV cure, elite controllers have been proven to have a unique genetic makeup, a potent T cell response, and keep HIV at bay in ‘gene deserts’, regions of the chromosomes which prevent the virus from being transcribed. These traits differentiate elite controllers from progressors of HIV [101–103].

Intriguingly, studies indicate a higher proportion of elite controllers are females, underscoring the importance of investigating gender-specific factors in HIV research [101]. In the case of Loreen Willenberg, often referred to as the San Francisco patient, researchers sampled over 184 million CD4 + T cell genomes from her body and were unable to find any replication-competent or intact HIV DNA [104]. Similarly, the Esperanza patient from Argentina, diagnosed in 2013, was on ART only briefly during her two pregnancies. Despite this, no intact or replication-competent virus has been detected in her body [105]. These cases highlight the potential role of sex-specific immune responses in achieving functional cures and the need for further research into the underlying biological mechanisms. Therefore, the underrepresentation of women in HIV clinical trials may prevent crucial information in developing effective treatments for everyone affected by HIV.

## Conclusions

Finding a definitive cure for HIV remains one of the most complex issues in modern medicine. EBT-101 marks a significant advancement, yet recent clinical trials reveal the complexities of translating preclinical success into clinical outcomes, with viral rebound after ART interruption. Addressing these complexities necessitates a multifaceted approach that incorporates genetic diversity, epigenetic regulation, post-transcriptional modifications, chromatin remodeling, and sex-specific factors, as summarized in Table 1.

**Table 1:** Inclusion and exclusion criteria

Aspect			Implications for Therapy
Genetic Diversity	Drug Resistance and Mutations	HIV exhibits significant genetic diversity, including multiple subtypes (A, B, C, D, etc.) and recombinant forms (CRFs, URFs).	Tailor ART regimens based on subtype-specific resistance profiles to enhance treatment efficacy and manage drug resistance.
		Different subtypes have unique resistance profiles and mutation patterns, influencing ART efficacy.	Incorporate comprehensive DRM screening in ART management to identify and counteract resistance early, ensuring effective treatment regimens and minimizing drug resistance.
	Geographic and Research Bias	Studies, primarily done on subtype B, have limited information on resistance mutations in other subtypes. This may result in less effective treatments for regions where non-subtype B variants are predominant.	Expand research efforts to include diverse subtypes prevalent in non-Western regions to that ART regimens are effective globally.
Epigenetics and HIV Latency	Epigenetic Role and HIV Integration	Epigenetic modifications like histone deacetylation and DNA methylation play a crucial role in maintaining HIV latency by inhibiting viral transcription.	Therapeutic strategies should address both epigenetic changes and integration-induced disruptions.
		HIV integration disrupts host gene expression, causing genomic instability and impacting cellular processes.	
	Histone Deacetylase Inhibitors (HDACis), PKC Agonists, and Other LRAs	HDAC inhibitors (HDACis) target histone deacetylases to reverse HIV latency in CD4 + T-cells by activating HIV transcription, which can lead to increased viral protein expression and potentially enable the immune system to clear infected cells or induce HIV-1-mediated cell death. But, they have had limited success in reducing latent HIV reservoirs.	Effective strategies should combine agents targeting different aspects of latency and address the diverse mechanisms of HIV reservoir maintenance.
		PKC agonists, including prostratin and bryostatin, effectively trigger latent HIV expression by activating transcription factors like NF-κB, which bind to the HIV LTR region and initiate HIV mRNA transcription. But, they have little effect on polyadenylated or multiply spliced transcripts.	
Post-Transcriptional Modifications	Epitranscriptomics	m^6 A modifications are prevalent in HIV RNA, affecting viral replication.	m^6 A modifications can impact the efficacy of therapies targeting viral replication, including cART and CRISPR-based editing. Disruption of m^6 A modifications could reduce viral replication and influence drug response. Targeting m^6 A pathways might enhance therapy efficacy or lead to novel treatments.
		Modifications in the Rev response element (RRE) enhances Rev protein binding, aiding viral RNA export and replication.	
Chromatin Remodelling	Mechanisms of Chromatin Modulation and Latency	Histone H3 lysine 9 trimethylation (H3K9me3) is crucial for gene repression and HIV-1 latency, as it is rapidly added to unintegrated HIV-1 DNA after nuclear entry, leading to the silencing of viral genes and complicating gene therapy approaches.	Understanding and targeting chromatin modulation mechanisms can help develop therapies to reactivate latent HIV-1 reservoirs.
		Vpr protein can induce degradation of HDACs (HDAC1 and HDAC3), affecting chromatin structure and latency.	Combining approaches targeting histone modifications and using tools like CRISPR to enhance Vpr expression may improve therapy outcomes.
		Histone acetylation, regulated by histone acetyltransferases (HATs) and histone deacetylases (HDACs), involves adding or removing acetyl groups to histone tails, affecting chromatin structure and gene expression by either opening or compacting the chromatin.	
	LRAs and their Ineffectiveness	LRAs aim to reactivate latent HIV-1 but face challenges due to the complexity of HIV-1 signalling pathways.	Combining LRAs targeting different signalling pathways and chromatin modifications may be necessary to achieve comprehensive reactivation of latent HIV-1 reservoirs.
		The effectiveness of LRAs is limited by heterogeneous reservoirs.	
		The variability in chromosomal integration sites also impacts HIV phenotypic expression, contributing to LRA limitations.	
Sex-based Disparities and Hormonal Influence	Biological Differences, Hormones and Disease Progression	Female HIV infection often shows a more robust early immune response, with higher CD4 + T cell counts, and lower viral loads compared to males.	Gender-specific differences in immune responses should be considered in developing HIV treatments.
		Males typically exhibit higher viral loads and lower CD4 + T cell counts.	Investigating the characteristics of elite controllers, especially the higher proportion of females, can provide insights into potential gender-specific mechanisms for HIV control.
		Hormones like oestrogen and progesterone influence the female genital tract’s mucosal membranes and immune responses.	
		Oestrogen enhances mucosal barriers and immune responses, potentially reducing HIV susceptibility compared to progesterone-dominated periods.	
		Elite controllers are individuals who maintain undetectable viral loads and stable CD4 + T cell counts without ART. A higher proportion of elite controllers are female, suggesting potential gender-specific factors in HIV control and cure.	
	X Chromosome and Immune Response	Females have two X chromosomes, with incomplete inactivation in some cells, which can offer an immunological advantage.	Understanding the role of X-linked genes can help develop targeted therapies that leverage these genetic advantages.
		TLRs on the X chromosome, such as TLR-7, are involved in detecting viral components and initiating immune responses.	

With diverse subtypes and recombinant forms, the genetic variability of HIV presents profound implications on treatment efficacy and disease progression. For example, a different treatment strategy might need to be considered for those with fast-progressing subtypes. The genetic heterogeneity of HIV, with its various subtypes and recombinant forms, emphasizes the need for a personalized approach to HIV treatment. This strategy is especially important for fast-progressing subtypes, which may respond differentially to ART. A personalized treatment strategy that considers pharmacogenomic parameters, such as host genetic variability, can improve ART efficacy and reduce side effects. For example, the HLA-B5701 screening test is a notable pharmacogenomic technique in clinical practice for identifying patients at risk of hypersensitivity to the antiretroviral medication abacavir [106]. Research also indicates that other host genetic factors, including sex, BMI, heredity, and genetic markers, impact ART efficacy and toxicity, leading to variable treatment outcomes among patients [107]. Integrating these pharmacogenomic insights with knowledge of specific HIV subtypes could enhance treatment outcomes, potentially reducing mortality and improving quality of life for individuals, especially those with fast-progressing forms of the virus.

Additionally, it is crucial to have a nuanced understanding of the intricate mechanisms governing epigenetic regulation, particularly chromatin remodeling and RNA modifications. These mechanisms play a pivotal role in elucidating HIV latency, viral replication dynamics, and immune evasion strategies. Given these findings, adding epigenetic profiling into HIV treatment regimens represents a possible path towards personalized therapy. Patients with high levels of m6A alterations may benefit from approaches that reverse these modifications, increasing ART efficacy by reactivating latent HIV and making it more susceptible to immune clearance. This strategy might be strengthened by using m6A inhibitors in addition to traditional ART, which would reactivate latent HIV reservoirs by increasing viral protein expression, making it easier for the immune system to detect and eradicate the virus [108]. Furthermore, developing small molecule inhibitors that target the writers, erasers, and readers involved in the m6A modification process may boost treatment outcomes by modifying the epigenetic mechanisms that affect HIV latency [109].

Furthermore, sex-specific biological and hormonal variances distinctly affect susceptibility, immune responses, and treatment outcomes. Given these findings, a promising therapeutic approach could involve using estrogen receptor modulators, such as agonists or antagonists, to enhance ART outcomes for women living with HIV. For instance, the modulation of estrogen receptors, particularly ERα, has shown potential in reducing HIV replication and adjusting immune responses. Research suggests that ERα can activate antiviral pathways, potentially explaining sex-specific changes in viral load and immunological responses. Targeting estrogen receptor signaling may be a novel approach to addressing these sex-specific variations, with the goal of improving treatment efficacy and reducing HIV persistence [110, 111]. The success of estrogen receptor antagonists and agonists could be incorporated into future therapies. Similarly, lessons learned from elite controllers suggests that ensuring that there is a higher rate of women representation in trials might provide more clues to an HIV cure. To effectively tackle these challenges, a holistic and inclusive approach to HIV research and treatment is paramount. By integrating genetic, epigenetic, and sex-specific considerations into clinical trial designs, a better development of personalized and equitable therapeutic interventions can be achieved. This comprehensive understanding of HIV will serve as the cornerstone for innovative strategies that cater to the diverse needs of affected populations, advancing the collective effort in the global fight against HIV/AIDS.

## Data Availability

No datasets were generated or analysed during the current study.

## Abbreviations

AAV: Adeno-associated virus
ART: Antiretroviral therapy
ATP: Adenosine triphosphate
AZT: Azidothymidine
BACH2: BTB and CNC homology 2
BTB domain: Broad-complex, tramtrack, and bric-à-brac domain
CAB: Cabotegravir
CART: Combination antiretroviral therapy
CCR5: C-C chemokine receptor type 5
CDK9: Cyclin-dependent kinase 9
CNC homology: Cap’n’collar homology
CRF: Circulating recombinant form
CRISPR: Clustered regularly interspaced short palindromic repeats
DIS: Dimerization initiation site
DNA: Deoxyribonucleic acid
DNMTs: DNA methyltransferases
DRACH: D = A/G/U, R = A/G, A = adenine, C = cytosine, H = A/C/U (m6A RNA modification motif)
DRMs: Drug resistance mutations
FDA: Food and drug administration
GAG: Group-specific antigen
H3k9me3: Histone 3 lysine 9 trimethylation
HATs: Histone acetyltransferases
HDAC: Histone deacetylase
HDACis: Histone deacetylase inhibitors
HDACs: Histone deacetylases
HEK293T: Human embryonic kidney 293T cells
HIV: Human immunodeficiency virus
HMTis: Histone methyltransferase inhibitors
IFNα: Interferon alpha
JAK/STAT: Janus kinase/signal transducer and activator of transcription
LEN: Lenacapavir
LRA: Latency-reversing agent
LTR: Long terminal repeat
MAPK/ERK: Mitogen-activated protein kinase/extracellular signal-regulated kinase
m6A: N6-methyladenosine
METTL3/14: Methyltransferase like protein 3/14
mRNA: Messenger RNA
mTOR: Mechanistic target of rapamycin
NVP: Nevirapine
NF-κB: Nuclear factor kappa B
PBS: Primer binding site
PDCS: Plasmacytoid dendritic cells
PI3K/AKT/mTOR: Phosphoinositide 3-kinase/protein kinase b/mechanistic target of rapamycin
PKC: Protein kinase C
p-TEFb/CDK9: Positive transcription elongation factor b/cyclin-dependent kinase 9
PREP: Pre-exposure prophylaxis
PTM: Post-translational modification
RNA: Ribonucleic acid
RNAP-II: RNA polymerase II
RRE: Rev response element
RPV: Rilpivirine
RT: Reverse transcription
SIV: Simian immunodeficiency virus
ssRNA: Single-stranded RNA
TGF-β: Transforming growth factor beta
TLRs: Toll-like receptors
VPR: Viral protein R
VPR BP: VPR binding protein
WNt/β-Catenin: Wnt signaling pathway/beta-catenin
ZfPB-362-VPR: Zinc finger protein binding to VPR
